# Characterization of Atherosclerotic Plaque Coating for Thrombosis Microfluidics Assays

**DOI:** 10.1007/s12195-021-00713-9

**Published:** 2021-10-27

**Authors:** M. F. A. Karel, T. P. Lemmens, B. M. E. Tullemans, S. J. H. Wielders, E. Gubbins, D. van Beurden, S. van Rijt, J. M. E. M. Cosemans

**Affiliations:** 1 Department of Biochemistry, Cardiovascular Research Institute Maastricht (CARIM), Maastricht University, PO Box 616, 6200 MD Maastricht, The Netherlands; 2 MERLN Institute for Technology-Inspired Regenerative Medicine, Maastricht, The Netherlands

**Keywords:** Atherosclerosis, Atherothrombosis, Flow assay, Oxygen plasma treatment, Platelets, Spin coating, Thrombus formation

## Abstract

**Introduction:**

Studying arterial thrombus formation by *in vitro* flow assays is a widely used approach. Incorporating human atherosclerotic plaque material as a thrombogenic surface in these assays represents a method to model the pathophysiological environment of thrombus formation upon plaque disruption. Up until now, achieving a homogeneous coating of plaque material and subsequent reproducible platelet adhesion has been challenging. Here, we characterized a novel method for coating of plaque material on glass coverslips for use in thrombosis microfluidic assays.

**Methods:**

A homogenate of human atherosclerotic plaques was coated on glass coverslips by conventional manual droplet coating or by spin coating. Prior to coating, a subset of coverslips was plasma treated. Water contact angle measurements were performed as an indicator for the hydrophilicity of the coverslips. Homogeneity of plaque coatings was determined using profilometric analysis and scanning electron microscopy. Thrombogenicity of the plaque material was assessed in real time by microscopic imaging while perfusing whole blood at a shear rate of 1500 s^−1^ over the plaque material.

**Results:**

Plasma treatment of glass coverslips, prior to spin coating with plaque material, increased the hydrophilicity of the coverslip compared to no plasma treatment. The most homogeneous plaque coating and highest platelet adhesion was obtained upon plasma treatment followed by spin coating of the plaque material. Manual plaque coating on non-plasma treated coverslips yielded lowest coating homogeneity and platelet adhesion and activation.

**Conclusion:**

Spin coating of atherosclerotic plaque material on plasma treated coverslips leads to a more homogenous coating and improved platelet adhesion to the plaque when compared to conventional droplet coating on non-plasma treated coverslips. These properties are beneficial in ensuring the quality and reproducibility of flow experiments.

**Supplementary Information:**

The online version contains supplementary material available at 10.1007/s12195-021-00713-9.

## Introduction

Atherothrombotic events, including myocardial infarction and stroke are still major causes of death in the western world.^17,20^ Plaque disruption by either erosion or rupture leads to exposure of plaque constituents to the flowing blood, resulting in thrombus formation that can lead to vessel occlusion. The pivotal importance of platelets in atherosclerosis has been studied for many years, but still some underlying molecular mechanisms and the effectiveness of novel (combined) therapies are ill-defined.^2^

Over the past few years there has been an increasing interest to study thrombus formation using *in vitro* flow assays. With this technique whole blood is perfused over fibrillar collagen through a channel at predefined shear rates. Collagen represents the damaged vessel wall and the channel a simplified blood vessel. Such microfluidics systems enable studying platelet adhesion, activation, and thrombus formation with or without coagulation at predefined shear stresses. Advantages and limitations of *in vitro* flow models for assessing thrombus formation are discussed in several excellent reviews, e.g. by the Scientific and Standardization Committee (SSC) on Biorheology of the International Society on Thrombosis and Hemostasis.^10^

Main thrombogenic components of the human atherosclerotic plaque include collagen type I and tissue factor (TF).^3,6,13,19,21^ These thrombogenic components are commercially available and are used to mimic the damaged atherosclerotic plaque. However, atherosclerotic plaque-collagen is reported to structurally differ from collagen of healthy connective tissue, which may alter platelet reactivity upon vascular damage.^7,13,19^ To improve mimicking of pathophysiological processes of arterial thrombosis in *in vitro* flow assays, human atherosclerotic plaque tissue can be used as a thrombogenic surface.

In addition to our group,^3^ only two other groups worked on the development of an *in vitro* model for thrombus formation incorporating atherosclerotic plaque material.^13,19^ Either cross sections of coronary arteries with atherosclerosis,^19^ cross sections of atherosclerotic plaques^1^ or crushed/homogenized atherosclerotic plaques^1,3,13^ were used. In the case of applying crushed/homogenized atherosclerotic plaques, a droplet of this material was pipetted on a glass coverslip.

The main difficulty in achieving a stable and homogenous adhesion of homogenized atherosclerotic plaque material onto a glass surface is the hydrophobic character of glass. It is well known that oxygen plasma treatment can increase the wettability of glass resulting in even distribution of water across the surface.^16^ We aimed to determine whether the effects of oxygen plasma treatment improved the coating of human atherosclerotic plaque material on glass. In addition to the use of oxygen plasma treatment we included spin coating of the plaque material, as it is a common technique for applying thin homogeneous coatings on a flat surface. When a solution is spun at high speeds, the surface tension of the fluid and the centripetal force create an even distribution over the surface.

In this article, we tested the hypothesis that plasma treatment with spin coating technique results in more homogeneous plaque surface and leads to more platelet adhesion when compared to the conventional droplet coating method.

## Materials and Methods

### Blood Drawing

Blood was obtained from healthy volunteers free from antiplatelet and anticoagulant medication for at least two weeks. All healthy volunteers gave full informed consent for participation according to the Helsinki declaration. Blood was collected into 3.2% trisodium citrate tubes (Greiner Bio-one), the first 3 mL were discarded.

### Preparation of Human Plaque Tissues

Permission was obtained from the local Medical Ethics Committee (Maastricht University). Four advanced atherosclerotic plaques were collected at autopsy from carotid artery and used in compliance with institutional guidelines (Department of Pathology, Academic Hospital Maastricht), as previously described.^3^ Plaque parts were immediately frozen into liquid nitrogen and stored at − 80 °C. After thawing, tissues were homogenized in PBS pH 7.45 by sonication (3 mm probe, 12 microns peak to peak, MSE, Nuaillé, France). As heat can be generated by sonication, the glass tube containing the plaque tissues was placed in ice during sonication. The obtained homogenates were centrifuged three times at 2240 g for 10 min to obtain cell- and lipid free homogenates. Pellets were resuspended in sterile PBS at a concentration of 16.5 mg tissue wet weight/ml. Collagen concentration in the plaque homogenate was measured using Sircol insoluble collagen assay according to the manufacturer’s instructions (Biocolor, Carrickfergus, United Kingdom). Factor Xa activity in the homogenate was kinetically measured using CS-11(65) (Hyphen BioMed, Mason, OH, USA). In short, plaque homogenate was diluted in HNBSA/Ca^2+^ buffer containing 25 mM Hepes (pH 7.7 at room temperature), 175 mM NaCl, 5 mg/mL bovine serum albumin and 3 mmol/L CaCl_2_ and incubated for 20 min at 37 °C. Reactions were started by adding factor VIIa (0.72 nmol/L) (Novo Nordisk, Bagsvaerd, Denmark), factor X (60 nmol/L) (Innovative Research, Novi, MI, USA), and phospholipid vesicles (7.2 μmol/L, phosphatidyl choline:phosphatidyl serine, 80:20) (Avanti Polar Lipids, Delfzijl, the Netherlands). For reference, TF (Innovin, Siemens Healthcare, Marburg, Germany) was used.

### Preparation of Microspot Coating

According to our standard operating protocol glass coverslips (24 × 60 mm #1, Thermo-Fisher, Waltham, MA) were cleaned and degreased in 1M hydrochloric acid (HCl) in 50% ethanol and washed twice with milli-Q water, then coated with either 2 *µ*L microspots of Horm collagen type I (50 *µ*g/mL, Takeda Austria GmbH, Linz, Austria) or 2 *µ*L human plaque material (16.5 mg/mL wet weight) in a humid atmosphere, referred to as our standard ‘droplet coating’ method. Here, we tested two modifications to our coating protocol: plasma pretreatment of glass coverslips and spin coating of plaque material instead of droplet coating. For plasma treatment, after standard cleaning and degreasing, the glass coverslip surface was activated with hydroxyl groups by oxygen plasma treatment (PCCE plasma machine Diener electronic GmbH + Co. KG). Plasma was applied for 1 min at 0.40 mbar and a power output of 70 W. Subsequently, a microspot of 2 *µ*L human plaque material (16.5 mg/mL) was coated on the glass coverslip for 12 min (droplet method) or a thin homogeneous coating of human plaque material was created using a SCK-300P/S Spin Coater (Instras Scientific LLC, Ridgefield Park, NJ). For the latter, due to practical reasons, a total volume of 4 *µ*L plaque homogenate (16.5 mg/mL wet weight) was spun over the glass coverslip at spinning speeds of 700 rpm for 10 s followed by 2550 rpm for 30 s. For a subset of experiments, presented in Suppl. fig. 3, a plaque concentration of 3.3 mg/mL wet weight was used for spin coating. After coating, all coated glass coverslips were immediately blocked with HEPES buffer pH 7.45 (136 mM NaCl, 10 mM HEPES, 2.7 mM KCl, 2 mM MgCl_2_) containing 1% BSA. Coverslips were then mounted onto a transparent flow chamber (height 50 *µ*m, width 3.0 mm, length 30 mm), air-tight clamped into a holder and pre-rinsed with HEPES buffer (additional 0.1% glucose and 0.1% BSA), allowing for whole blood perfusion over the coated surface.

### Whole Blood Perfusion Assay

An established whole blood microfluidics assay in a multi-parameter setting was applied for a detailed assessment of alterations in thrombus formation under physiological conditions, as described.^4^ An image of the experimental setup used can be found in Supplemental fig. 4. The citrate-anticoagulated blood was recalcified by adding 40 *µ*M PPACK (Milipore), 7.5 mM CaCl_2_ and 3.7 mM MgCl_2_ within a minute prior to perfusion. Where indicated, blood samples were pre-incubated with 1 *µ*g/mL tirofiban (Aggrastat), for 10 min. Whole blood was perfused for 5 min at an arterial shear rate of 1500 s^−1^. Brightfield images were taken every minute at the same spot to monitor platelet adhesion and thrombus formation during whole blood perfusion. Whole blood perfusion was followed by rinse buffer perfusion (1 U/mL heparin and 2mM CaCl_2_ in HEPES buffer pH 7.45) during which brightfield and fluorescent images were taken at multiple spots throughout the chamber. All samples were measured within two hours after blood drawing. Image recording was performed with an EVOS fluorescence microscope. Representative brightfield and fluorescence microscopic images were captured using an Olympus 60× oil-immersion objective and a GFP 470 nm LED diode cube. Images were collected at 8 bits (1360 × 1024 pixels). Blind analysis was performed. Based on brightfield images of adhered platelets, the percentage of surface area covered with platelets (surface area coverage, SAC) was assessed using ImageJ (1.53c) as described previously.^15^ In addition, morphological score, contraction score, and multilayer score were determined by visual inspection of the platelet features per microspot in comparison to preset reference images. The following criteria were used: morphological score (range 0–5): 0, no or hardly any adhered platelets (< 15 platelets/microscopic image); 1, multiple single-adhered platelets (> 15 platelets/microscopic image); 2, extensive coverage of single-adhered platelets (monolayer); 3, small platelet aggregates; 4, intermediate platelet aggregates; and 5, large-size platelet aggregates. Contraction score (range 0–3): 0, no contraction, single platelets; 1, little contraction, initial aggregate formation; 2, medium contraction, loose aggregates; 3, close contraction, compact stable aggregates. Multilayer score (range 0–3): 0, no platelet layers; 1, one to two platelets in height; 2, layers of > 2 platelets in height but still able to identify single platelets; 3, multilayered aggregates, unable to identify single platelets.^15^

### Scanning Electron Microscopy and Profilometer

Scanning electron microscopy (SEM) images were taken of the coated coverslips. Coatings were fixed in 3.7% glutaraldehyde and dehydrated using a series of increasing alcohol concentrations. Slides were then put in hexamethyldisilane and sputter coated in gold and silver. Images were taken using a Teneo scanning electron microscope (FEI, US).

Surface measurements to estimate film thickness were performed using a confocal laser scanning microscopy (VK-X series Keyence, Japan). Measurement mode was set to surface profile with a 20X lens magnification and measurement size set to super fine (2048 × 1536). Analysis of images obtained were carried out using Keyence MultiFileAnalyzer software.

### Water Contact Angle Measurement

Water contact angle (WCA) of glass substrates was measured with the sessile drop technique at room temperature using a contact angle goniometer (Drop shape Analyzer DSA25, Kruss, Germany). For this, plasma treated and non-plasma treated ‘control’ coverslips, both cleaned and degreased in 1M HCl in 50% ethanol and washed twice with milli-Q water, were fixed on a stage of the goniometer. A droplet of 5 *µ*L water was dropped onto the coverslips and the contact angle was determined after 1 min.

### Statistics

Statistical analyses were performed with GraphPad Prism 9 (GraphPad Software, San Diego, CA). Statistically significant differences (*p* < 0.05) compared to controls were tested with a Mann-Whitney test, a Kruskal-Wallis test, or a two-way ANOVA, as appropriate.

## Results

### Combination of Plasma Treatment and Spin Coating Results in a Homogeneous Distribution of Human Plaque Material

**Figure 1:**
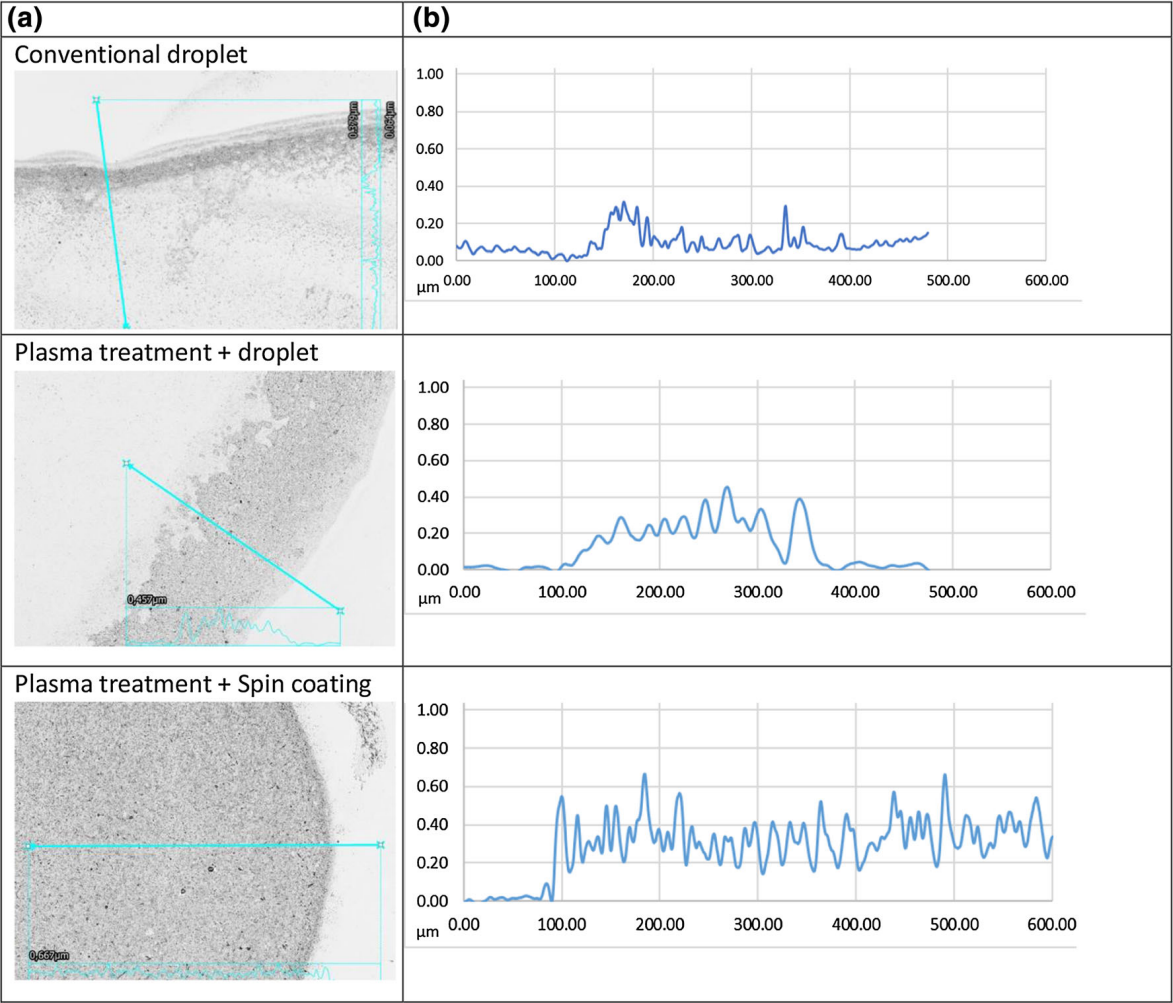
Laser profile image at 20× (a) and line profile measurement (b) of plaque coating using; conventional droplet method, plasma treated coverslip with droplet and plasma treated coverslip with spin coating.

Homogenates were coated on the glass using four different methods, (1) the droplet method, (2) additional plasma treatment of the glass coverslip before droplet method, (3) plasma treatment of the glass coverslip combined with spin coating of the plaque homogenate and (4) spin coating of the plaque homogenate without plasma treatment. Thus, the variables in the experimental setup were plasma treatment of the glass coverslip (yes/no) and application of the plaque material via manual pipetting (droplet method) or spin coating. Spin coating without plasma treatment (method 4) resulted a spiral shaped, uneven, distribution of the plaque material. This can be explained by the hydrophobic nature of the glass coverslip which caused the droplet to spin in the same direction as spun by the spin coater. Because of this uneven coating, we decided to exclude this condition for further experiments. When treated with plasma, the glass substrate became much more hydrophilic (57.5 ± 1.1° vs 2.1 ± 1.1°), as determined with a contact angle goniometer (Suppl. fig. 1). The diameter of a spin coated plaque spot (± 20 mm) was considerably larger than when using the droplet coated method (2mm), providing us with a larger surface area for flow experiments. However, it remains challenging to determine the amount of plaque material per *µ*m^2^ because of other contributors next to the total surface area, such as the hydrophilicity of the coverslip and the homogeneity of the plaque coating. We investigated the homogeneity of the coated plaque material through profilometer measurements, which allows assessment of coating homogeneity and thickness (Fig. 1, Suppl. table 1), and used scanning electron microscopy (SEM) to assess surface morphology (Suppl. fig. 2). Profilometer analysis indicated that the area roughness parameters average step height and root mean square height of the coating obtained upon plasma treatment and spin coating were higher than with conventional droplet coating (0.36 vs. 0.17 and 0.28 vs. 0.11 *µ*m, respectively). This can potentially be attributed to a more uneven distribution of the droplet coated plaque material versus spin coated plaque material. Indeed, plaque material accumulates at the edge of the spot or appeared to be washed away in the center upon droplet coating (Fig. 1a). Moreover, line profile measurements showed a more homogeneous surface when using the spin coating technique versus droplet coating (Fig. 1b). Lastly, visual inspection of SEM images also revealed homogeneous plaque coating upon plasma treatment of the coverslip and spin coating of plaque material (Suppl. fig. 2).

### Plasma Treatment Combined with Spin Coating Led to Increased Platelet Deposition on Human Plaque Material Versus Droplet Coating Without Plasma Treatment

**Figure 2:**
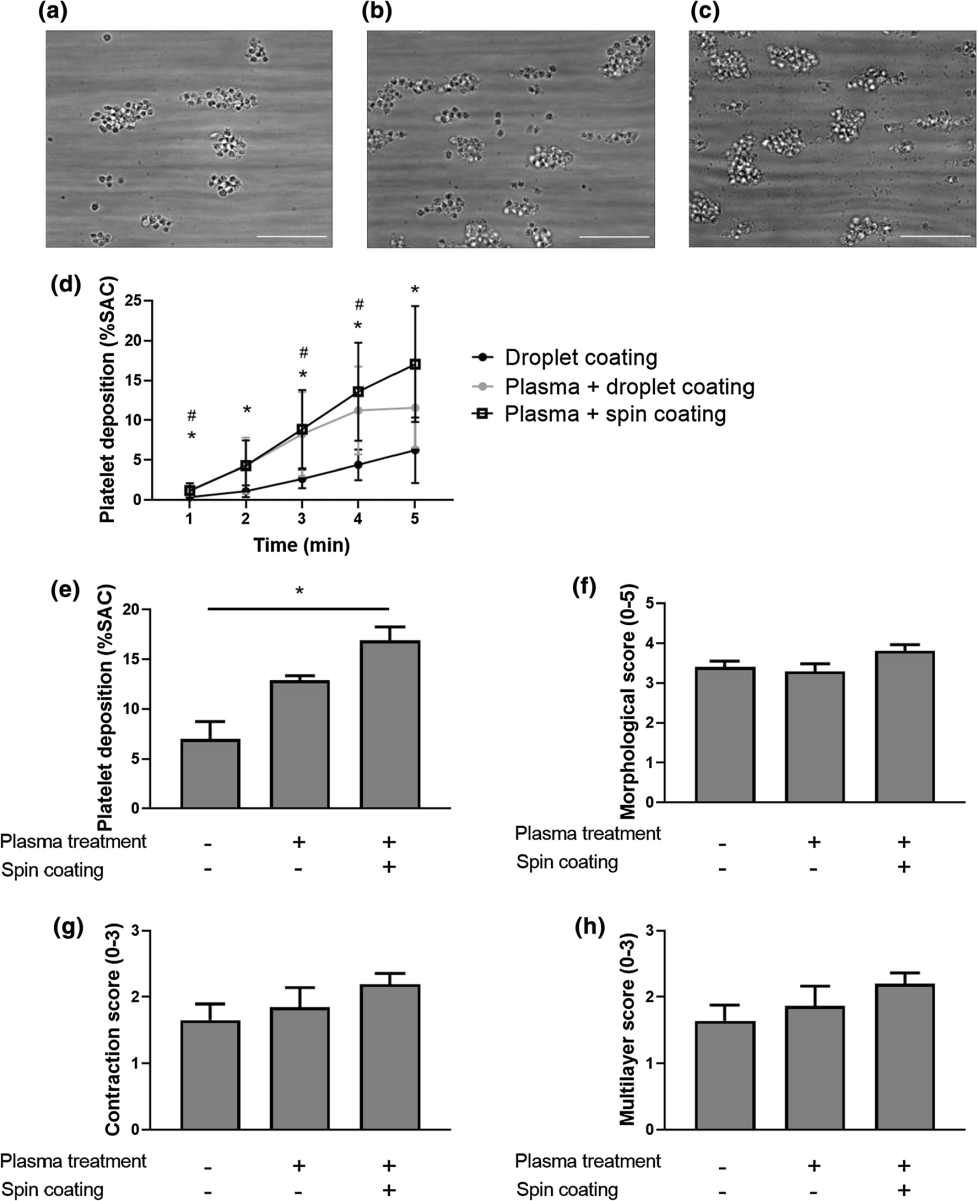
Coating human plaque material after plasma treatment and combined with spin coating increased platelet deposition. (a–c) Representative brightfield images after 5 min of human whole blood perfusion at an arterial shear rate of 1500 s^−1^ at room temperature. Human plaque material was coated through (a) droplet method, (b) plasma treatment combined with the droplet method, (c) plasma treatment combined with spin coating. Thrombus parameters include (d) platelet deposition (% SAC) in time, (e) platelet deposition (% SAC) after 5 min, (f) thrombus morphological score (0–5), (g) thrombus contraction score (0–3) and (h) thrombus multilayer score (0–3). Scale bar 25 *µ*m. Mean and SEM, *n* = 4–7, *p < 0.05 for plasma bonding + spin coating vs droplet method and ^#^*p* <0.05 for plasma bonding + droplet method vs droplet method, Kruskal–Wallis test and two-way ANOVA.

To assess the effect of different coating methods on platelet deposition of human plaque material, a series of *in vitro* flow assays was conducted (Fig. 2). Knowing that collagen and tissue factor are the main thrombogenic components in plaque material,^3,6,13,19,21^ we first determined the collagen- and tissue factor concentration. The plaque homogenate used in this study contained 27.6 *µ*g/mL collagen and had a factor Xa activity that corresponded to 4.7 pM TF. We assumed that an increase in adhesion of biologically active plaque material to the coverslip corresponds to an increase in platelet deposition. Human whole blood from healthy volunteers was recalcified and perfused over atherosclerotic plaque material at a calculated arterial wall shear rate of 1500 s^−1^ for 5 min. All pre-analytical variables (e.g., blood, buffers, perfusion time, shear rate, imaging, and analysis) were the same throughout the different conditions, with the coating technique for atherosclerotic plaque material as only exception. Platelet count and hematocrit level were within the normal range for all samples. An increase in platelet deposition was seen on plaque material coated via plasma treatment + droplet (+83%, *p* = 0.39) or via plasma treatment + spin coating (+141%, *p* = 0.01) when compared to droplet coating without plasma treatment (Fig. 2e). A similar positive trend was observed for thrombus growth in time (Fig. 2d) and when thrombi were scored for morphology, contraction, and multilayer (Figs. 2a–2c and 2f–2h). Plasma treatment by itself had no binding properties for platelets (data not shown). When plaque concentration was diluted five times to 3.3 mg/mL (Suppl. fig. 3), and coated through plasma treatment combined with spin coating it had a similar platelet deposition (6.13 ± 0.59 %, vs 7.01 ± 1.72 %, mean ± SEM), morphological score (3.47 ± 0.23, vs 3.41 ± 0.15), contraction score (1.76 ± 0.27, vs 1.65 ± 0.24) and multilayer score (1.76 ± 0.27, vs 1.64 ± 0.24) when compared to the droplet method (Suppl. fig. 3). Thus, plasma treatment combined with spin coating led to increased platelet deposition on human plaque material versus droplet coating without plasma treatment and droplet coating with plasma treatment.

### Temperature has no Influence on Platelet Deposition and Thrombus Characteristics on Plaque Surfaces

**Figure 3:**
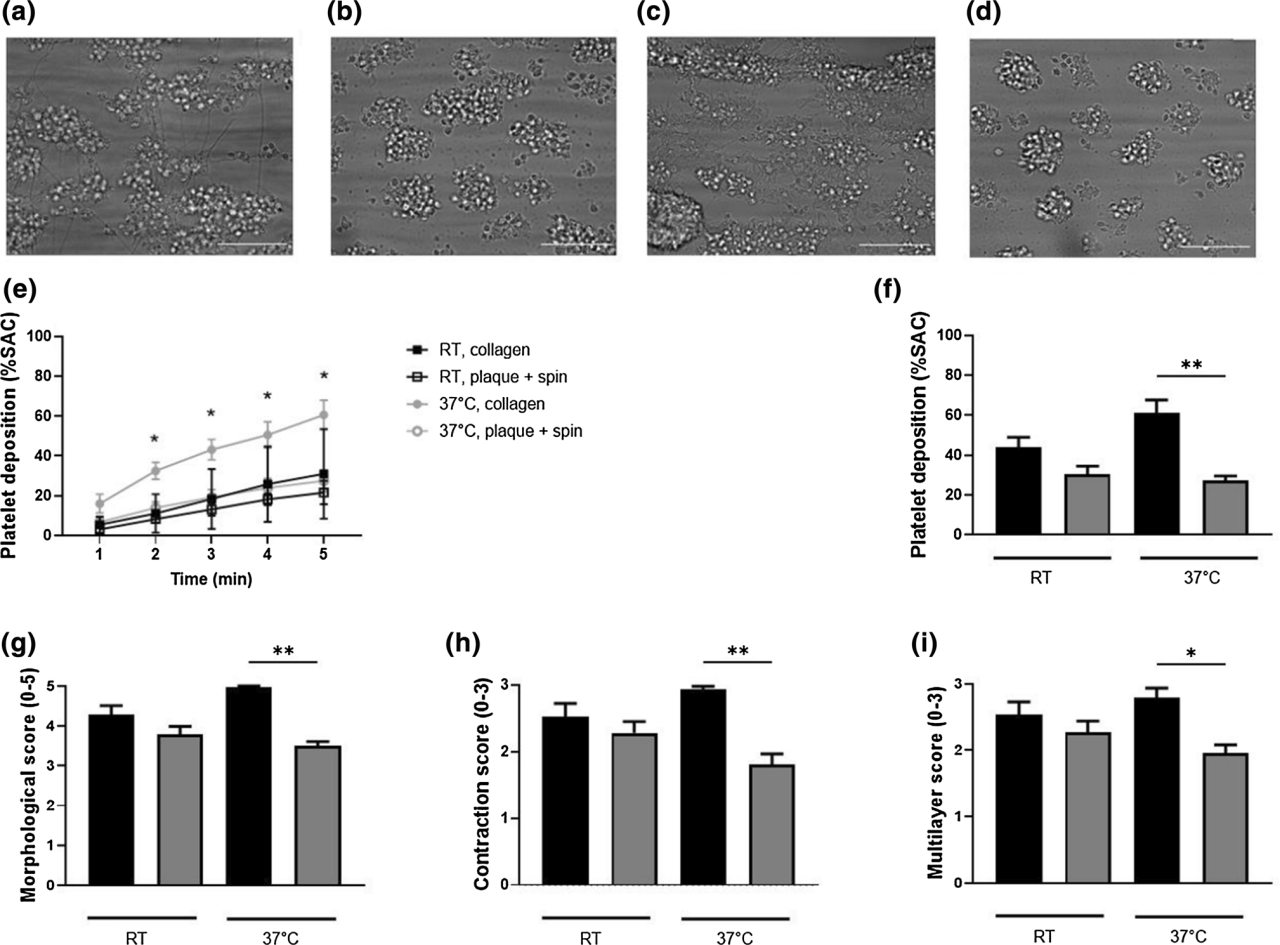
Temperature has no influence on platelet parameters on plaque surfaces. Human whole blood was perfused over collagen or plaque material at an arterial shear rate of 1500 s^−1^ at either room temperature or 37 °C. Horm collagen type I (black bars) was coated through droplet method at a concentration of 50 *μ*g/mL. Human plaque material (grey bars) was coated through plasma treatment combined with spin coating at a concentration of 16.5 mg/mL. Local platelet deposition was assessed by brightfield microscopy in time. (a–d) Representative brightfield images after 5 min of blood perfusion. Flow assay temperature and thrombogenic surface were as follows: (a) room temperature, collagen coating, (b) room temperature, plaque coating, (c) 37 °C, collagen coating, and (d) 37 °C, plaque coating. Thrombus parameters including (e) platelet deposition (% SAC) in time, (f) platelet deposition (% SAC), (g) thrombus morphological score (0–5), (h) thrombus contraction score (0–3) and (i) thrombus multilayer score (0–3) were measured after 5 min of blood perfusion. Scale bar 25 *µ*m. Mean and SEM, *n* = 5, *p < 0.05, **p < 0.01, Kruskal–Wallis test and two-way ANOVA.

To assess a possible effect of temperature during perfusion on thrombus formation we compared performing perfusion experiments at room temperature (RT) versus at 37 °C (Fig. 3). Human whole blood from healthy volunteers was recalcified and perfused over either collagen or atherosclerotic plaque material that was coated through plasma treatment combined with spin coating, at a wall shear rate of 1500 s^−1^ for 5 min. Representative images of formed thrombi on a collagen surface at RT (Fig. 3a) and 37 °C (Fig. 3c) and a plaque surface at RT (Fig. 3b) and 37 °C (Fig. 3d) are shown. We observed no difference in platelet deposition (collagen: *p* = 0.48, plaque: *p* > 0.99) and thrombus characteristics (morphology (collagen: *p* = 0.78, plaque: *p* > 0.99), contraction (collagen: *p* = 0.68, plaque: *p* = 0.83) and multilayer score (collagen: *p* > 0.99, plaque: *p* > 0.99) when comparing RT to 37 °C (Figs. 3e–3i). However, when comparing collagen to plaque surface at RT there was a trend towards larger, more contracted, and higher thrombi on the collagen surface, and this trend became more pronounced when measured at 37 °C (Figs. 3f–3i).

### Combination of Plasma Treatment and Spin Coating can be Used to Study Platelet Inhibitors

**Figure 4:**
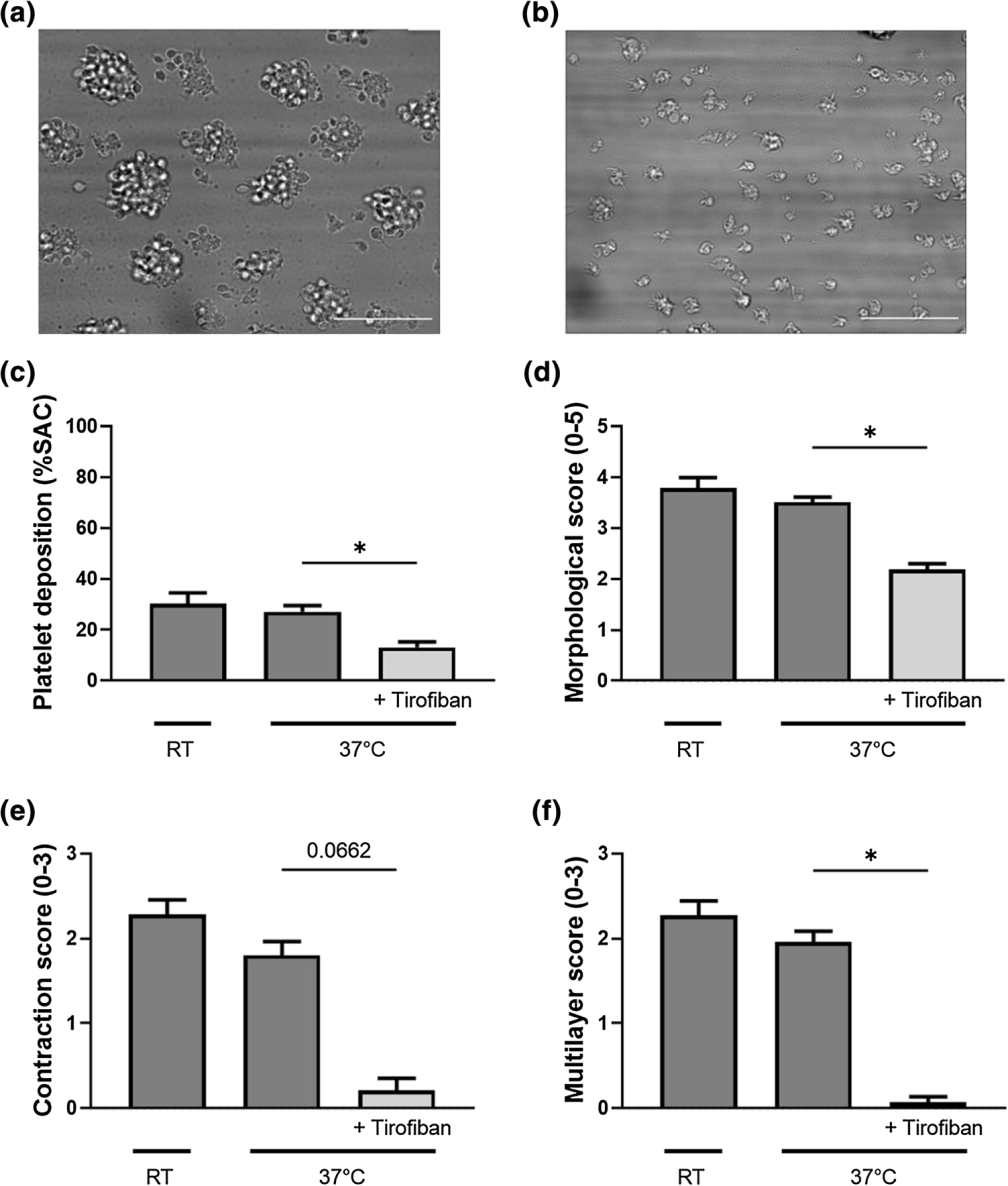
Platelet deposition on plaque material is decreased when inhibited with tirofiban. Human whole blood was perfused over human plaque material at a shear rate of 1500 s^−1^ at room temperature and 37 °C (with or without tirofiban). (a, b) Representative brightfield images after 5 min of blood perfusion at 37 °C with (b) and without (a) tirofiban. (c) Local platelet deposition (%SAC), (d) thrombus morphological score (0–5), (e) thrombus contraction score (0–3) and (f) thrombus multilayer score (0–3) were assessed after 5 min of blood perfusion by brightfield images. Scale bar 25 *µ*m. Mean and SEM, *n* = 4–5, *p < 0.05, Kruskal–Wallis.

To determine if our novel model was also suitable to test inhibitors, we examined the role of the glycoprotein IIb/IIIa inhibitor Tirofiban in plaque-induced thrombus formation of human blood under flow conditions. Pre-incubation with tirofiban resulted in decreased platelet deposition by 52% (*p* = 0.02670), smaller (morphological score, *p* = 0.04), less contracted (contraction score, *p* = 0.07) and lower (multilayer score, *p* = 0.001) thrombi (Fig. 4). Thus, inhibition of plaque-induced thrombus formation can be measured in our *in vitro* flow assay.

## Discussion

In this study we demonstrated that plasma treatment of glass coverslips resulted in a more homogenous coating of human atherosclerotic plaque material and led to increased platelet deposition on the plaque material when compared to no plasma treatment. In addition, spin coating of the plaque material, instead of droplet coating, further increased platelet deposition.

Previous studies demonstrated that collagen in atherosclerotic plaques structurally differs from collagen of healthy connective tissue, which may alter platelet reactivity upon vascular damage.^7,13,19^ To improve the pathophysiological relevance of arterial thrombosis in *in vitro* flow assays, human atherosclerotic plaque tissue can be used as a thrombogenic surface. Previously we and Reininger *et al.* have reported a variance in platelet deposition between plaque samples.^3,14^ We used snap-frozen fresh lipid-rich carotid plaque material, which according to Penz *et al.* is the optimal protocol for storing human atherosclerotic plaque material.^12^ The atheromatous plaque material containing the cap was gently homogenized and pooled, allowing exposure of all thrombogenic plaque components to flowing blood in a flow chamber.

Plasma treatment of glass can both clean and activate a glass surface, improving the wettability -as shown in the present study by water contact angle measurements- and the adhesion of proteins like collagen and fibronectin.^5,22^ Rendering glass substrates hydrophilic before plaque coating will also work using other methods, such as with piranha solution or hydroxy silanes. To our knowledge, few reports have directly compared these methods. One study did report elevated hydroxyl groups on the plasma treated surfaces relative to piranha chemically treated surfaces, which effected later glass treatment steps with polymers.^11^ Hence, comparing different approaches to render glass hydrophilic in the setting of in vitro thrombosis studies could yield valuable information regarding coating of thrombogenic material for thrombosis studies. Spin coating is used in a wide variety of industries and technology sectors. The primary advantage of spin coating over other methods is its ability to produce uniform coatings quickly and easily. Spin coating parameters that affect the thickness of the coatings include surface tension, viscosity, and rotation rate. 3D laser scanning confocal microscope imaging of the surface coating confirmed a smoother and more homogenous distribution of plaque material when plasma treatment of the glass coverslip and spin coating of the atherosclerotic plaque material were combined. Average height of our coating after plasma bonding and spin coating was 0.36 μm, which is thinner compared van Zanten *et al.* (6 *μ*m thick cross sections of arteries)^19^ and Reininger *et al.* (< 5 *μ*m homogenized, pooled atherosclerotic plaques).^14^ An increase in coating thickness at the edge of the spot after spin coating is a common phenomenon as fluid moves outward there is a higher evaporation rate at the center compared to the edge (non-uniform evaporation rate) causing a small coating thickness difference.^8,18^ This effect was least pronounced when using a combination of plasma treatment and spin coating technique. With this method we could obtain a larger surface area for flow experiments, reducing any effects of the edge of the coating on outcome parameters.

We showed a significant increase in platelet deposition, which is indirectly correlated to an increase in plaque material adhesion, when using plasma treatment in combination with spin coating. Platelet deposition was comparable to Busygina *et al.* who coated plaque homogenates at an unknown concentration (pooled, from 5 patients) and perfused hirudinized blood for 5 min at 1500 s^−1^.^1^ We previously established the intra- and inter-individual variation in thrombus formation parameters on collagen type I using the same microfluidics device as in the present study and blood from six healthy donors. The inter- and intra-individual coefficients of variation for morphological score were both 0% and, respectively, 24.1 and 10.5% for platelet deposition,^4^ indicative of the robustness of in vitro microfluidics assays. To foster comparison between studies, we advise to report at least the following variables: (1) the source (vessel), histopathological classification and processing of the plaque material (homogenization, storage); (2) the concentration of the plaque material, collagen content, and—in case of flow experiments under coagulant conditions—tissue factor concentration; (3) dimensions (width, height) of the flow chamber channel; (4) the type of anticoagulant used and, if applicable, method used for recalcification; and (5) the analysis of thrombus surface (image analysis). As pre-analytical variables, including donor status (i.e. food and medication intake prior to donating), blood drawing methodology and duration of blood storage could influence platelet deposition,^9^ it is advised to keep these variables the same as much as possible. See an excellent review by the SSC on Biorheology for more detailed insight in pre-analytical variables and recommendations for standardisation.^9^ It should be noted that although collagen type I is the main platelet activating component in human plaque material, the plaque-collagen concentration does not correlate with the platelet activating potential of the plaque.^13^ Up till now it is not possible to quantify the thrombogenicity of plaque material. Determining platelet adhesion and thrombus formation is an indirect measurement for plaque-thrombogenicity.

To improve mimicking of pathophysiological processes in *in vitro* flow models even further, temperature should also be considered as an additional parameter. We explored the effect of temperature on platelet parameters on both collagen and atherosclerotic plaque coating and observed increased platelet deposition on collagen versus plaque material when measuring at body temperature (37 °C) but not at room temperature. No significant difference of platelet adhesion to plaque material was observed between RT and 37 °C. These results do not exclude a potential role for temperature when studying platelet deposition on different thrombogenic substrates with(out) inhibitors.

In conclusion, we have described a new method for coating atherosclerotic plaque material that results in more homogenous coating and improves platelet adhesion and activation. These properties are beneficial in ensuring the quality and reproducibility of flow experiments. This method can be easily implemented in future experiments that aim to look at the mechanisms behind thrombus formation on plaque material or the antithrombotic properties of novel antiplatelet compounds.

## Supplementary Information

Below is the link to the electronic supplementary material.

Supplementary file1 (DOCX 3285 kb)

## Abbreviations

SAC: Surface area coverage
SSC: Scientific Standardization Committee
SEM: Scanning electron microscopy
TF: Tissue factor
