# Supplementary material for: Characterization of Atherosclerotic Plaque Coating for Thrombosis Microfluidics Assays
Source: Cell Mol Bioeng. 2021 Oct 27;15(1):55–65. doi: 10.1007/s12195-021-00713-9 (PMC8761191; doi:10.1007/s12195-021-00713-9)

**Supplemental Table and Figures**

**Characterization of atherosclerotic plaque coating for thrombosis microfluidics assays**

Karel M.F.A.^1*^, Lemmens T.P. ^1*^, Tullemans B.M.E.^1^, Wielders S.J.H. ^1^, Gubbins E. ^2^, van Beurden D. ^2^ van Rijt S. ^2^ Cosemans J.M.E.M.^1^

*Equal contribution

Department of Biochemistry, Cardiovascular Research Institute Maastricht (CARIM), Maastricht University, Maastricht, the Netherlands^1^. MERLN Institute for Technology-Inspired Regenerative Medicine, Maastricht, the Netherlands^2^.

Abbreviated title: atherosclerotic plaque coating technique for flow assay

Correspondence: Judith M.E.M. Cosemans, Department of Biochemistry, Cardiovascular Research Institute Maastricht (CARIM), Maastricht University, PO Box 616, 6200 MD Maastricht, The Netherlands. E-mail: judith.cosemans@maastrichtuniversity.nl.

**Supplemental Table**

**Supplemental table 1:** Surface roughness parameters of coated plaque material, as determined by profilometric analysis.

|  | Conventional droplet method | Plasma treatment + droplet method | Plasma treatment + spin coating method |
| --- | --- | --- | --- |
| Average step height (µm) | 0.17 | 0.22 | 0.36 |
| Root mean square height (Sq) (µm) | 0.11 | 0.20 | 0.28 |

**Supplemental Figure legends**

**Supplemental figure 1: Water contact angle of untreated versus plasma treated glass substrates measured by a sessile drop technique at room temperature using a contact angle goniometer.** Photographs of a water droplet on an untreated (A) and a plasma treated (B) glass coverslip. Red line indicates the contact angle and the green line the droplet contour.

**Supplemental figure 2: Characterization of plaque deposition after plasma treatment and spin coating using SEM**. Representative SEM images are depicted with 3500x zoom. **A)** Plasma treatment combined with spin coating of atherosclerotic plaque material **B)** Plasma treatment combined with manual pipetting (droplet method) of atherosclerotic plaque material **C)** Conventional method with manual pipetting of atherosclerotic plaque material (droplet method).

**Supplemental figure 3: Decreased platelet deposition on diluted human plaque material.** Human plaque material was coated through spin coating after plasma bonding of the glass coverslip. Plaque material was coated at a concentration of either 16.5 mg/mL (grey bars) and 3.3 mg/mL (white bars). Human whole blood perfusion at an arterial shear rate of 1500s^-1^ at room temperature. **A-B)** Representative brightfield images after 5 minutes of blood perfusion. Human plaque material was coated at a concentration of **A)** 16.5 mg/mL or **B)** 3.3 mg/mL. Thrombus parameters include **C)** platelet deposition (% SAC) in time, **D)** platelet deposition (% SAC), **E)** thrombus morphological score (0-5), **F)** thrombus contraction score (0-3) and **G)** thrombus multilayer score (0-3) after 5 minutes. Scale bar = 25 µm. Mean and SEM, n = 4-7, *p < 0.05, Kruskal-Wallis test.

**Supplemental figure 4: A photograph of the experimental setup used for the whole blood perfusion assay.**

**Supplemental Figures**

**Supplemental figure 1** Karel *et al.*


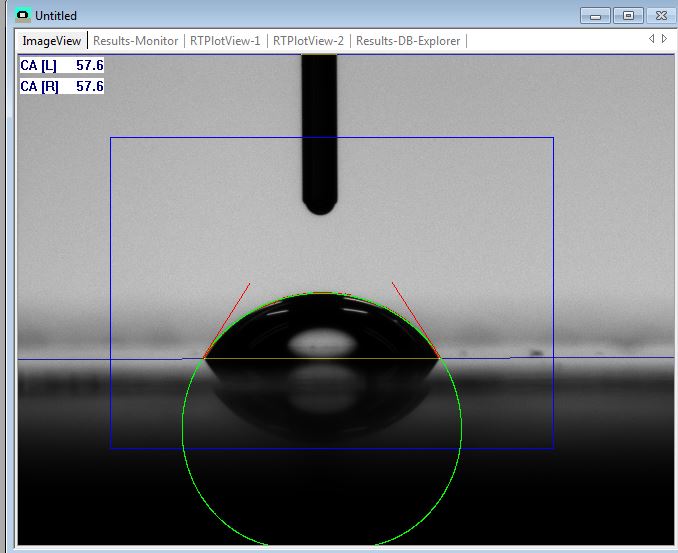

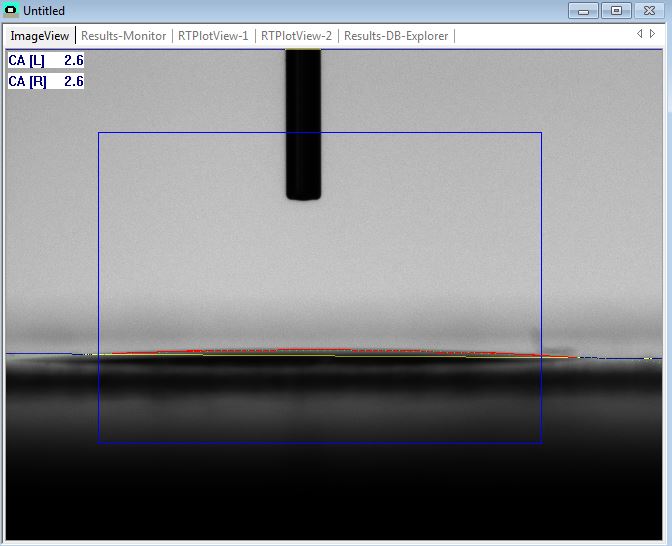


A B

**Supplemental figure 2** Karel *et al.*


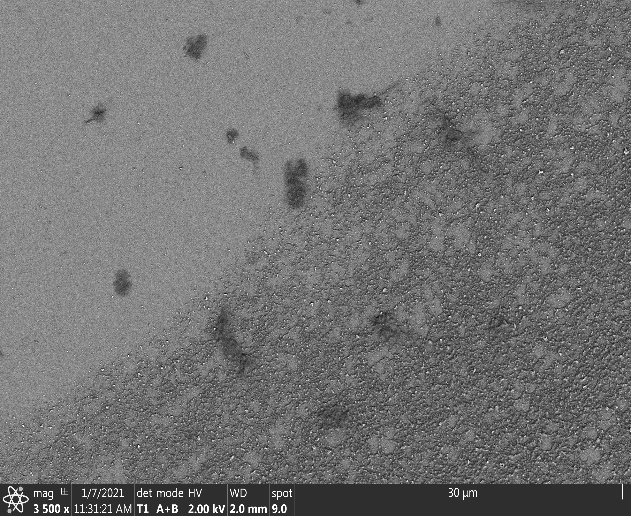

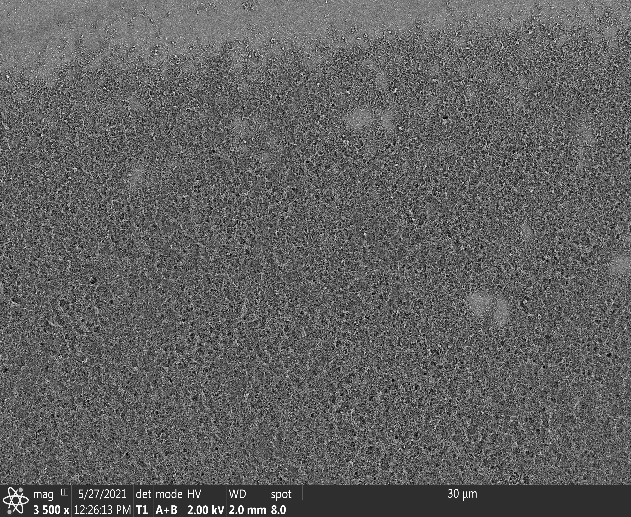

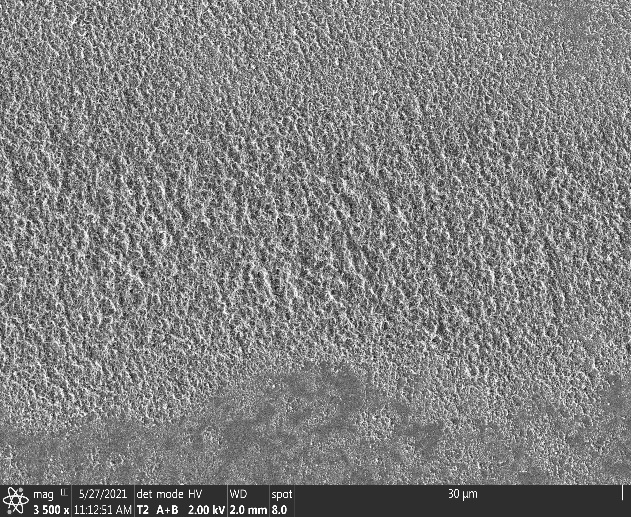


A

B

C


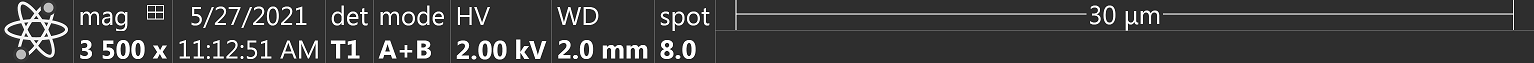


**Supplemental figure 3** Karel *et al.*


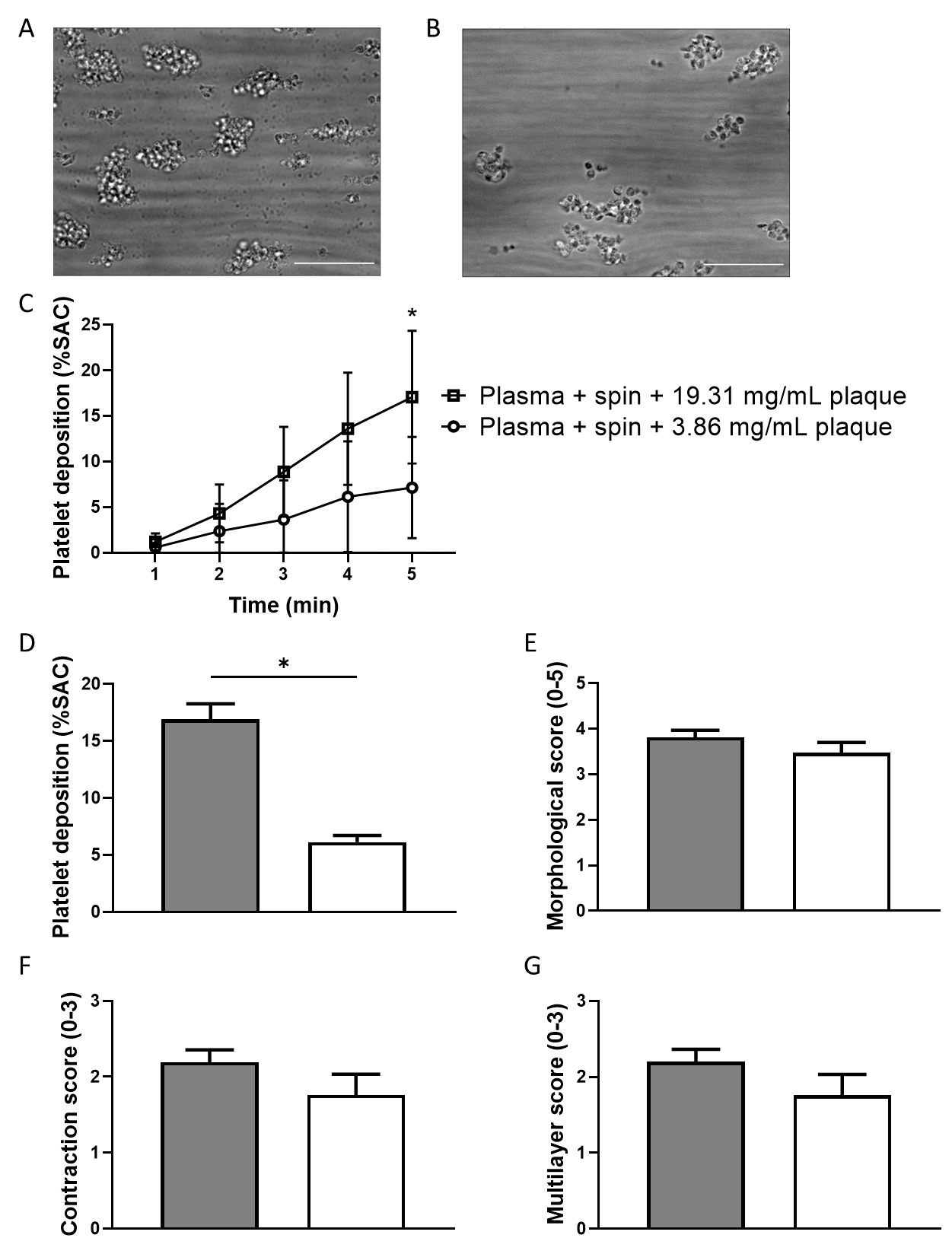


**Supplemental figure 4** Karel *et al.*


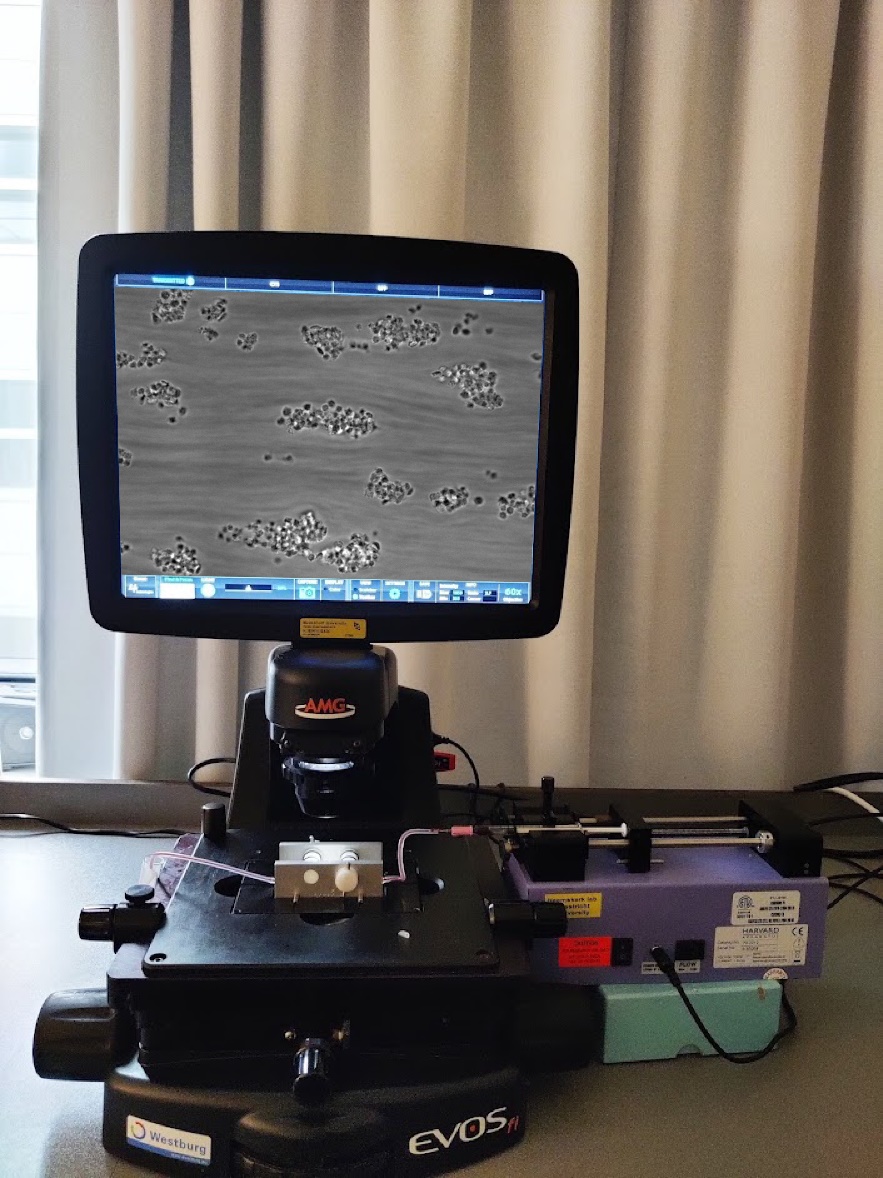

Supplement: Supplementary file 1 — Supplementary file1 (DOCX 3285 kb) [file 12195_2021_713_MOESM1_ESM.docx]
